# Predictors for Psychological Distress 2 Months After Mild Traumatic Brain Injury

**DOI:** 10.3389/fneur.2019.00639

**Published:** 2019-06-18

**Authors:** Eirik Vikane, Kaia Frøyland, Hanne Langseth Næss, Jörg Aßmus, Jan Sture Skouen

**Affiliations:** ^1^Department of Physical Medicine and Rehabilitation, Haukeland University Hospital, Bergen, Norway; ^2^Centre for Clinical Research, Haukeland University Hospital, Bergen, Norway; ^3^Department of Global Public Health and Primary Care, University of Bergen, Bergen, Norway

**Keywords:** mild traumatic brain injury, predictors, psychological distress, anxiety, depression, outcome, treatment

## Abstract

**Objective:** To predict psychological distress at 2 months for patients with mild traumatic brain injury.

**Method:** A prospective cohort study of 162 patients with mild traumatic brain injury (MTBI) admitted consecutively to an outpatient clinic at Haukeland University Hospital, Norway. Demographic data were obtained from Statistics Norway and injury characteristics were obtained from the hospital records. Sick leave data from the last year before the injury were obtained from The Norwegian Labor and Welfare Service. Self-report questionnaires were used to obtain history about earlier disease and symptom profiles. The Hospital Anxiety and Depression Scale (HAD) detecting states of depression and anxiety were used as the dependent variable in a stepwise linear regression. Pre-injury factors and injury-related factors were examined as potential predictors for HAD.

**Results:** In the first steps we observed a significant association between HAD at 2 months and education, whiplash associated disorder (WAD), and earlier sick listed with a psychiatric diagnosis. In the final step there was an association only between HAD and self-reported anxiety and WAD. There were no associations between HAD and injury-characteristics like severity at Glasgow Coma Scale or intracranial injury.

**Conclusion:** Patients with low education, earlier psychiatric diagnosis, self-reported earlier anxiety and WAD were more likely to develop a psychological distress after a MTBI. These findings should be taken into consideration when treating patients with MTBI.

## Introduction

Mild traumatic brain injury (MTBI) is a major public-health concern, and more than 600 patients per 100,000 people are suffering a MTBI (1, 2). The annual incidence of hospital-treated MTBI is around 100–300 patients per 100,000, and in Norway <100 patients per 100,000 are hospitalized (1, 3).

Common acute symptoms are headache, fatigue, dizziness and cognitive impairment associated with pain, sleep disturbance and psychological distress (4, 5). For the majority of patients with a MTBI the symptoms resolve, but between 5 and 20% develops post-concussion symptoms (PCS) lasting more than 12 months (4, 6–11).

PCS are more common among MTBI-patients compared to other patients suffering a non-head trauma (4, 12). PCS can be divided into somatic, cognitive, and emotional complaints (13). Somatic complaints include headache, dizziness, nausea, fatigue, problems with vision, noise sensitivity, and sleeping problems. Cognitive symptoms include problems with memory or concentration and reduced speed of processing. Emotional symptoms include depression, anxiety, frustration, and irritability (6, 14).

There is a debate in the existing literature whether PCS result from organic injury in the brain, psychological factors or both (15–17). Some studies demonstrate that PCS are associated with pre-injury mental and physical health, injury-related stress and early post-injury cognitive impairment (4, 17). Other authors have found an association between stress, depression, anxiety, all-or-nothing behavioral, and negative expectations about recovery with the development of PCS (18, 19). It is stated that the development and maintenance of PCS can best be explained by a biopsychosocial model, including both neurobiological, psychological and social factors (20). This model that includes pre-injury factors can also explain the multifactorial etiology of persistent symptoms after MTBI (21).

Despite the favorable outcome of MTBI for the majority of patients, a substantial group of patients report symptoms and disability after MTBI. To improve the outcome, several authors have suggested a planned follow-up visit after MTBI to screen for specific treatable conditions, such as depression, anxiety, or other modifiable factors like expectations or coping strategies (22–25). Clinical guidelines, like those from Ontario, Canada recommend a rehabilitation model involving an early evaluation of signs and symptoms combined with education focusing on the normalization of symptoms and reassurance of the expected favorable outcome within 3 months (26). There is a consensus that treatment should be based on a biopsychosocial model and for a gradual return to daily activities and work (26). The guidelines recommends that somatic, cognitive, or behavioral difficulties should be treated symptomatically, and that a management strategy for each symptom including treatment for mental health disorders must be considered (26). It is important to identify characteristics of patients who are at risk of developing psychological distress like anxiety or depression after a MTBI. To improve research about the outcome after a traumatic brain injury (TBI) common data elements are developed, where several pre-defined variables are potential predictors for a functional outcome after a TBI (27). In addition, both pre-injury and early post-injury psychological factors are important predictors for the functional outcome after a MTBI (28, 29). Zahniser et al. found that early psychological distress after MTBI appeared to function as a precursor to functional impairment, and one study for MTBI patients aged 6–17 years found an association between a pre-injury diagnosis of anxiety and psychological distress after a MTBI (30, 31). To the best of our knowledge, pre-injury and injury-related factors have not been investigated as predictors of psychological distress after a MTBI among adults.

The objective of this study was to identify which clinical characteristics predict psychological distress at 2 months after injury for patients with MTBI.

## Methods

### Patients and Settings

This is a prospective cohort study with 162 patients admitted to a planned clinical follow-up within 2 months after a MTBI at the Department of Physical Medicine and Rehabilitation at Haukeland University Hospital, Norway after a MTBI. All patients hospitalized for 5 h or longer at the Department of Neurosurgery at Haukeland University Hospital, Bergen, Norway, from January 2009 to July 2011, with an ICD-10 diagnosis of S06.0–S06.9 received a planned follow-up by a rehabilitation specialist 6–8 weeks after the injury at a multidisciplinary outpatient clinic. After finishing the planned follow-up, the sick-listed patients at an age 16–55 years were recruited to a randomized clinical trial. The aim of the main multicenter study was to find out if a specific multidisciplinary model improved return to work (32). Pre- injury-, injury-, and post-injury-related clinical variables were analyzed to find any significant associations with psychological distress 2 months post-MTBI.

### Inclusion Criteria

In accordance with the Task Force on MTBI and the American Congress of Rehabilitation Medicine, MTBI was defined as a Glasgow Coma scale (GCS) measure of 13–15 within 30 min or the lowest score during the first 24 h post-injury, unconsciousness for <30 min and posttraumatic amnesia <24 h (33, 34). The participants lived in a mixed rural and urban community, and the majority of them were Norwegian residents (Caucasians). Patients attending the follow-up session, fitting the inclusion criteria and providing a written informed consent were consecutively recruited to the study.

### Exclusion Criteria

We omitted patients on disability pension or unemployed in the last 6 months. Patients with a severe head trauma or other diseases that had a significant impact on working skills were excluded. Other exclusion criteria included the following: lack of informed consent, lack of Norwegian language skills or a history of substance abuse in the medical records.

### Procedures

When discharged from neurosurgical service for clarity, MTBI patients received an information pamphlet about their MTBI and how to address their symptoms. They were also informed that they would receive a planned follow-up consultation within 2 months post-injury. The participants received a self-report questionnaire and an appointment with a specialist in physical and rehabilitation medicine 6–8 weeks post-injury. At follow-up a clinical interview and an examination was performed with reassurance of an expected favorable outcome after the injury. Patients meeting the inclusion criteria were then offered to participate in the study.

The Self-report questionnaires collected 6–8 weeks post-injury were used to obtain patients history about and screening for PCS, psychological distress, disability, and pain. Demographic data were obtained from the self-report questionnaire and information about education and income from Statistics Norway. Injury characteristics including acute CT scan were obtained from the medical records during the patient's emergency stay. Data regarding sick leave and diagnosis the last year before the injury were obtained from a national register, the Norwegian Labor and Welfare Service (NAV). After 16 days on sick leave, every citizen in Norway are paid by NAV. The majority of patients were given a diagnosis and received their sick-leave certificate by a general practitioner, a minority by medical specialists. In addition, patient with a musculoskeletal disorder may be sick-listed for up to 12 weeks by a manual therapist or chiropractor.

An accredited third-party agency, Statistics Norway, linked the clinical data with the sick leave data from NAV.

### Measures

Psychological distress measured with Hospital Anxiety and Depression Scale (HAD) at 6–8 weeks post-MTBI was used as the main outcome and was the dependent variable in a stepwise linear regression.

HAD is a self-reported questionnaire consists of 14 items assessing states of depression (seven items) and anxiety (seven items) (35). The patients rate each item using a four-point scale from 0 to 3: 0 = no symptoms during the last week; 3 = a severe symptom or symptoms most of the time during the last week. The HAD has been validated for traumatic brain injuries and documented to have high reliability (35, 36). The total sum of scores for HAD was used in the analyses. The subscale of anxiety and depression ranges from 0–21, 8–10 are mild cases and 11 are set as a cut-off for moderate or severe anxiety or depression (35, 37).

Pre-injury and injury-related factors were examined as potential predictors for psychological distress 2 months post-MTBI.

#### Pre-injury Factors

We obtained information about the income for 1 year (2010) from Statistics Norway, categorized above the mean or not. Pre-injury factors assessed from the self-report questionnaire consisted of age in years, sex, social status such as number of children still living with parents, education and employment status. Education was categorized either as primary education or secondary and higher with more than 10 years of education.

#### Injury-Related Factors

Injury mechanisms classified as traffic accidents, falls, violence and others (sports) were obtained from the self-report questionnaire. In addition, occupational injuries were also registered. The GCS, neurological status, seizures, headache, neck pain, whiplash associated disorder (WAD), findings on CT scan, alcohol intoxication and length of hospital stay were collected from the medical records from the emergency stay. Patients diagnosed with the ICD-10 diagnosis of S13.4 by a neurosurgeon at the emergency stay was defined as WAD, a non-medical term describing a sudden distortion of the neck. We defined those patients who did not undergo a CT as having no intracranial injury in the analysis, based on the information from the medical records. GCS, a clinical scale for assessing the depth and duration of unconsciousness and coma, was used to classify MTBI based on the first observed GCS within 30 min or the lowest score the during the first 24 h (38). In the preliminary analyses, findings on CT was categorized as type of bleeding, contusion, location of injury, intracranial injury or not, and fractures of the skull, face and neck. Length of post-traumatic amnesia (PTA) was measured using a standardized interview at the follow-up 6–8 weeks after the MTBI, asking the patients to retrospectively recall events. PTA was dichotomised into more or <1 h.

#### Pre-injury Factors Obtained From a National Registry and Retrospective Self-Rating Factors

We received information from the national registry NAV whether the participants had been sick-listed during the last year before injury and diagnosed with a psychiatric diagnosis, TBI, fatigue, attention deficit disorder, headache, neck pain, a musculoskeletal disorder, neurological disorders, or other diagnosis classified as other disease. The participants had to be sick-listed for more than 16 days to be registered in the national registry. In addition, the participants ticked off at the self-report questionnaire obtained 6–8 weeks post-injury if they at the pre-injury period had anxiety, depression, prior head injury, headache, neurological disorders, or other diseases.

Data registered in the study were entered into the database by two independent co-workers unfamiliar with the aim and content of the study. A biostatistician was responsible for performing and controlling the statistical analyses. The biostatistician was not involved in the treatment or collecting data.

### Statistical Methods

Data analyses was completed with IBM SPSS Statistics for Windows, Version 24.0. Armonk, NY: IBM Corp.

We used a linear regression model to assess the predictors for the total sum score for HAD. In the preliminary analyses we estimated the unadjusted model for each of the pre-injury and injury-related factors, to detect all predictors with an association to psychological distress. In the preliminary analyses the significance level was set to 0.10.

In the first step in the fully adjusted model we estimated all significant pre-injury predictors and injury-related predictors, where the significance level was set to 0.05.

In the second step we added pre-injury predictors about sick leave from the national registry the NAV in the fully adjusted model.

Finally, in the third step we estimated the fully adjusted model for all significant predictors from the first two steps and retrospective self-report data about pre-injury diseases.

Additionally, to take into account potential confounding and reflect all aspects of the study in the fully adjusted model, we ensured to have age and sex as essential properties of the cohort in the model.

We used pairwise deletion for missing data to ensure that we used all available data and achieve maximal power in the estimated models. The significance level was set to 0.05 for all analyses in the fully adjusted model.

## Results

As presented in an earlier published paper, we identified 343 patients with MTBI admitted consecutively to the Departments of Neurosurgery from January 2009 to July 2011 (39). Of these, 96 patients decided to not attend the planned follow-up 6–8 weeks post-MTBI basically due to a favorable outcome (39). In addition, 92 were not eligible to the study at 6–8 weeks follow-up; 45 substance abuse, 22 significant somatic disease, five significant psychiatric disease, and four lack of language. Finally, 171 patients were eligible to the study, nine declined to participate, and 162 patients were included in the analyses.

As given in Table 1, the median age was 33 years, and 63% of the participants were men. The majority of the injuries comprised a fall (47%). Regarding education, 38% have only primary school education with 10 years or less. A CT scan was performed for 94% of the patients and showed intracranial injury for 19% of the patients. GCS was 15 for 77% of the patients, and 12% reported PTA for more than 1 h. It was 27% of the patients who meet the criteria for anxiety at HAD with a score of eight or higher on the subscale, 15% meet the criteria for a depression and 12% had both anxiety and a depression. The mean score of the HAD was 8, 52, standard deviation 7, 30 and range from 0 to 31.

**Table 1:** Demographic data and clinical characteristics 6–8 weeks after mild traumatic brain injury.

**Variable**	**Total**	***n* (%)**
**Pre-injury factors**		
Age, years^a^	162	33 [16, 55]
Sex, men	162	102 (63%)
Education, primary school, 10 years or less of education	162	62 (38%)
Income in thousand NOK^a^	149	339 [1.3, 1,145]
**Injury-related Factors**		
Cause of injury	162	
Traffic accident		31 (19%)
Fall		76 (47%)
Assault		36 (22%)
Sports injury and others		19 (12%)
Glasgow Coma scale (GCS)^a,b^	162	15 [13, 15]
GCS 13		7 (4%)
GCS 14		30 (19%)
GCS 15		125 (77%)
Radiological examination^b^		
Intracranial injury (CT-scan)	162	30 (19%)
Frontal intracranial injury	162	24 (15%)
Whiplash associated disorder	162	18 (11%)
PTA > 1 h	92	11 (12%)
**Pre-injury factors from a national registry**		
Diagnosed during last year before injury		
Psychiatric diagnosis	162	18 (11%)
Headache and other neurological disorders	162	4 (3%)
Traumatic brain injury	162	2 (1%)
Musculoskeletal disorder	162	37 (23%)
**Pre-injury retrospective self-rating factors**		
Anxiety	162	21 (13%)
Depression	162	35 (22%)
Headache	162	24 (15%)
Neurological disorders	160	7 (4%)
Traumatic brain injury	161	31 (19 %)
Other disease	162	48 (30%)
**Psychological distress six to eight weeks post-injury**		
HAD anxiety score eight or higher	162	44 (27%)
HAD anxiety score 11 or higher	162	20 (12%)
HAD depression score eight or higher	162	24 (15%)
HAD depression score 11 or higher	162	11 (7%)

a *Median [min, max]*.

b *Measured at time of injury*.

The results of the linear regression analyses are given in Table 2. We abstain from presenting the non-significant results from the unadjusted model for each of the pre-injury and injury-related predictors presented in the measure section, which were not included in the fully adjusted models.

**Table 2:** Linear regression analyses of predictors in relation to psychological distress 2 months after mild traumatic brain injury.

	**Step 1**				**Step 2**			**Step 3**		
**Fully adjusted models, *N* = 147**	**N**	**B**	**CI (95%)**	***P*-value**	**B**	**CI (95%)**	***P*-value**	**B**	**CI (95%)**	***P*-value**
**Pre-injury factors**										
Age	162	0.11	(−0.0, 0.2)	0.069	0.08	(−0.0, 0.2)	0.194	0.09	(−0.0, 0.2)	0.104
Sex	162	−0.61	(−3.0, 1.8)	0.620	−0.75	(−3.1, 1.6)	0.522	−0.23	(−2.3, 1.9)	0.831
Education	162	**3.67**	**(1.0, 6.4)**	**0.008**	**3.40**	**(0.8, 6.0)**	**0.011**	2.17	(−0.2, 4.6)	0.074
Income	149	−2.73	(−5.5, 0.0)	0.053	−2.60	(−5.3, 0.7)	0.056	−2.11	(−4.5, 0.3)	0.086
**Injury-related factor**										
Whiplash associated disorder	162	**3.77**	**(0.7, 7.5)**	**0.046**	3.35	(−0.3, 7.0)	0.068	**3.55**	**(0.3, 6.8)**	**0.035**
Frontal intracranial injury	162	−2.23	(−5.5, 1.1)	0.183	−1.07	(−4.3, 2.2)	0.516	−1.22	(−4.1, 1.7)	0.405
**Pre-injury factors from a national registry**										
Sick-listed other diagnosis	162				1.80	(−1.1, 4.7)	0.223	1.84	(−0.8, 4.4)	0.164
Sick-listed musculoskeletal	162				1.69	(−1.2, 4.5)	0.242	0.76	(−1.8, 3.4)	0.564
Sick-listed psychiatric diagnosis	162				**5.29**	**(1.7, 8.9)**	**0.004**	2.04	(−1.6, 5.6)	0.263
**Pre-injury retrospective self-rating factors**										
Anxiety	162							**7.61**	**(3.6, 11.6)**	**<0.001**
Depression	162							1.88	(−1.6, 5.4)	0.289
Headache	162							1.12	(−1.8, 4.0)	0.446
Other disease	162							1.52	(−0.8, 3.8)	0.198

*Significance: p < 0.05 marked bold*.

In the first step in the fully adjusted model we observed in the linear regression model at a 5% significance level a significant association between HAD at 2 months and WAD and lower education. WAD had the largest beta value of 3.77 (0.7, 7.5) and education beta of 3.67 (1.0, 6.4). The pre-injury and injury-related variables explained 13% of the variance in psychological distress.

In the second step in the fully adjusted model we observed in the linear regression model a significant association between HAD at 2 months and lower education and a psychiatric diagnosis the last year before injury. A psychiatric diagnosis had the largest beta value of 5.29 (1.7, 8.9) and education a beta of 3.40 (0.8, 6.0). The pre-injury diagnosis from NAV explained an additional 8% of the variance in psychological distress.

In the final step in the fully adjusted model we observed a significant association between HAD at 2 months and pre-injury retrospective self-reported anxiety and WAD. Pre-injury self-reported anxiety had the largest beta value 7.61 (3.6, 11.6) and WAD a beta of 3.55 (0.3, 6.8). The R square for the final model was 0.396 with an adjusted R Square of 0.338, explaining an additional 19% of the variance in psychological distress.

There were no association between HAD and other injury-related measures like severity at Glasgow Coma Scale, PTA or intracranial injury in any of the steps in the adjusted models.

## Discussion

The aim of this study was to identify which clinical characteristics predict psychological distress at 2 months after a MTBI. Several variables predicted psychological distress at 2 months post-MTBI. However, in our final model, two variables contributed uniquely to psychological distress at 2 months, namely pre-injury retrospective self-reported anxiety and WAD.

Among pre-injury variables, lower education, psychiatric diagnosis the last year before injury and pre-injury self-reported anxiety were associated with the development of psychological distress after a MTBI. Singh et al. found an association between pre-injury and post-injury depression among more severe TBI-cases where 55% of the patients had a moderate or severe TBI (40). To our knowledge, earlier studies have not investigated predictors for psychological distress among adults after a MTBI. Among children and adolescents pre-injury anxiety, acute memory problems are associated with psychological distress at 4 weeks after a MTBI, and acute mental status predict psychological distress at 12 weeks (31). Among adults pre-injury depression is earlier demonstrated to be associated with post-injury anxiety and PCS (41). McCauley et al. found no association between sex, age, or education and the development of anxiety and PCS after a MTBI, equivalent to the lack of relationship between sex and psychological distress after a MTBI in our study (41). However, our findings are comparable with predictors for PCS after MTBI, where education and pre-injury mental health problems were among predictors for unfavorable outcome after a MTBI (4, 28, 42). Since psychological distress is a part of PCS, our findings support that PCS are associated with pre-injury mental health, emphasizing that PCS can best be explained by a biopsychosocial model and not as a result of neurobiological factors alone (4, 6, 14, 17, 20). In our study there were no association between pre-injury retrospective self-report of depression and the development of psychological distress post-injury, indicating the important role of pre-injury anxiety to explain the development of psychological distress after MTBI.

Several authors have found a non-linear association between age and outcome after a MTBI with a favorable outcome for patients aged 65 and older (28). In the main multicenter study the focus was to improve return to work. We therefore recruited only patients at the age between 16 and 55 years, which can explain the lack of association between age and outcome (32).

Among injury-related variables only WAD was associated with the development of psychological distress. In earlier literature it is described more pronounced psychological disorders among patients with chronic WAD (43). Further on, elements of anxiety like fear and catastrophizing are associated with the development of chronic WAD (44, 45). Finally, it is earlier demonstrated an association between elevated psychological distress after a motor vehicle crash and the development of MTBI and WAD (46). These findings may indicate an association between psychological distress and the development of both PCS and WAD after an injury. However, our finding is in accordance with earlier literature demonstrating an association between extracranial injuries and outcome after MTBI, indicating WAD as an indicator of the severity of the injury (42). In a biopsychosocial model the association between WAD and MTBI may then be a result of both psychological factors and the severity of the injury.

In this cohort there were no association between development of psychological distress at 2 months and other injury-related measures like severity at Glasgow Coma Scale, PTA or intracranial injury in any of the steps in the adjusted models. The impact of injury-related factor on PCS is debated, so far there is no consensus about the predicting value of MRI and the development of PCS (47). However, our findings are partly supported by studies that have found no association between the outcome after a MTBI and CT-abnormalities (28). In another study comparing a clinical model with adding a more advanced MRI brain morphometric characteristics to the model, the MRI-based measures had no additive value to predict outcome after MTBI (48).

It is noteworthy that several variables contributed to psychological distress at 2 months post-MTBI, including lower education, pre-injury psychiatric diagnosis, retrospective self-reported anxiety and WAD. However, only self-reported anxiety and WAD made a unique significant impact in our final model that explained 40% of the variance in psychological distress. Because our sample size was relatively small, care must be taken when interpreting the findings.

## Strengths and Limitations

The patients are recruited among the most severe MTBI-cases since they were hospitalized. A strength of this study was the use of clinical data about pre-injury diagnosis for sick-leave to avoid recall bias (49). However, short-term sick leave <16 days is missing, and we most likely have lost information about student sick-listed for <1 year, since students and unemployed must be on sick-leave for 1 year to receive any benefits from the NAV. In our study population 23 % were students.

However, a major limitation with our finding was that the strongest predictor for the development of psychological distress after a MTBI, was retrospective self-reported anxiety assessed in the self-report questionnaire 6–8 weeks post-injury, therefore these retrospective self-report predictors were added in step 3 in the model. According to earlier published studies self-reports may be more biased and less sensitive than more objective clinical characteristics (48). Patients reporting psychological distress after an injury, may be more likely to report earlier psychological problems at follow-up. In step 1 and 2 we avoided recall bias by using pre-injury data about sick leave from a national registry together with injury-related factors, where a pre-injury psychiatric diagnosis, education, and WAD predict the outcome. However, a major strength of this study was that both a pre-injury psychiatric diagnosis from the national sick-leave registry in step 2 and self-report anxiety in step 3 were associated with the development of psychological distress after a MTBI. To reduce the potential for selection bias, the guidelines from The Strengthening the Reporting of Observational Studies in Epidemiology (STROBE) statement was followed (50). By obtaining data from national registries, we improved the quality of the study and reduced the probability for data-collector bias.

However, we cannot exclude selection bias due to exclusion of patients out of work last 6 months before injury, and that potential participants did not attended the planned follow-up. According to an earlier publication from this cohort, the patients who did not attend the planned follow-up most likely had a favorable outcome and fewer needs for rehabilitation support (39). Many of the patients that were excluded from the study had a severe substance abuse, and was vulnerable to develop psychological distress after a MTBI (51). Our results cannot be transferred to this group of patients.

Another limitation is the assessment of clinical data in the medical records from the emergency hospital stay, where relevant information was missing such as the intensity of acute pain. CT scan was performed by 94% of the patients, which indicates that our results regarding intracranial findings are valid.

Acute emotional distress, coping style, and resilience are found to be associated with the outcome after a MTBI (28, 41). We have no information about coping style or resilience in our study, these factors may be important in predicting development of psychological distress after a MTBI. Further research should focus on determining which pre-injury factors including personal factors and coping style that have an association with the development of psychological distress after a MTBI, and implement these factors in future intervention studies. Our study may have some implications for rehabilitation after a MTBI. Clinically, a self-report questionnaire is easily administrated to screen for demographic data and psychological distress post-MTBI. Early detection of vulnerable patients with a thoroughly clinical interview about pre-injury mental health and offering them treatment may improve the outcome after a MTBI.

## Conclusion

Patients with low education, earlier psychiatric diagnosis, self-reported earlier anxiety and WAD were more likely to develop a psychological distress after a MTBI. These findings should be taken into consideration when treating patients with MTBI.

## Data Availability

All datasets generated for this study are included in the manuscript and/or the supplementary files.

## Ethics Statement

This study was carried out in accordance with the recommendations of The Norwegian Social Science Data Services, identifier NSD 20425 and the National Committees for Research Ethics in Norway. All subjects gave written informed consent in accordance with the Declaration of Helsinki.

## Author Contributions

EV and JS contributed the conception and design of the study. EV organized the database. EV and JA performed the statistical analyses and contributed to the section of statistics and results. EV wrote the first draft of the manuscript. EV, KF, HN, and JS contributed to manuscript revision, read and approved the submitted version.

### Conflict of Interest Statement

The authors declare that the research was conducted in the absence of any commercial or financial relationships that could be construed as a potential conflict of interest.
